# In-hospital sting challenge during venom immunotherapy has a high negative predictive value for subsequent sting reactions 

**DOI:** 10.5414/ALX02652E

**Published:** 2026-09-25

**Authors:** Nicola Wagner, Eva Oppel, Jarmila Gloning, Annett Walker, Sophia Czell, Franziska Ruëff

**Affiliations:** 1 Department of Dermatology, Uniklinikum Erlangen, Friedrich-Alexander-Universität, Erlangen-Nürnberg (FAU), Erlangen, and; 2 Department of Dermatology and Allergy, University Hospital, LMU Munich, Munich, Germany; *Equally contributing authors

**Keywords:** allergen immunotherapy, venom immunotherapy, venom allergy, Hymenoptera allergy, risk re-sting, anaphylaxis

## Abstract

Aims: Hymenoptera venom allergy is the most frequent elicitor of anaphylaxis in adults. Biomarkers predicting success of venom immunotherapy (VIT) are lacking. Only a re-sting may help to detect insufficiently protected individuals. The aim of the study was to investigate the prognostic value of a tolerated sting challenge with regard to future field stings. Materials and methods: A retrospective study included 585 consecutive patients of the AllergieZentrum Munich, Germany, who had tolerated a sting challenge test during VIT. Patients who were still on VIT were examined with a questionnaire evaluating the outcome of later field stings during ongoing VIT. Results: 439 of 585 patients (75%) could be evaluated. Of the 439 patients, 74/102 (75.2%) had tolerated a sting challenge with bee, 288/401 (74.1%) with yellow jacket, and 67/82 (81.7%) with both insects. In the follow-up time, 24.6% (108) had experienced at least 1 field sting. 104 of 108 patients (96.2%) had tolerated the sting without any reaction. Four patients (3.8%) reported systemic symptoms, 2 of whom experienced mild allergic immediate-type symptoms (1.9%). Conclusion: In patients on ongoing VIT a tolerated in-hospital sting provocation has a high negative predictive value (98.2%) in relation to the outcome of later field stings. The majority of these patients will subsequently not develop systemic reactions.

## Introduction 

Hymenoptera stings are the most frequent elicitor of severe anaphylactic reactions in adults [1]. Venom immunotherapy (VIT) is a highly effective therapy for patients with Hymenoptera venom allergy [2]. Some patients, however, may not be protected by standard treatment [3]. To identify this subgroup, it is recommended that all treated patients undergo a sting challenge test with a living insect [4]. A systemic allergic reaction at sting challenge or field sting will indicate treatment failure leading to immediate therapeutic consequences such as increasing the maintenance VIT dose. Thereby, a clinical protection may be achieved in the majority of patients [5]. 

Even if some biomarkers, e.g., IgG4 or the basophil activation test, change during the course of VIT [6, 7], there are still no reliable in-vitro parameters indicating successful therapy. A tolerated sting is the only method to verify success or failure of VIT. With respect to the predictive value after stop of VIT, the reliability of a tolerated sting challenge is higher than a tolerated field sting [8]. In contrast to challenge tests with other allergens, however, sting challenge tests are less standardized with a theoretical chance for falsely negative results. Limitations of sting challenge tests include a) uncertainties concerning the exact venom dose injected by the insect, b) seasonal variations of venom composition [9], and c) an inadequate design of the test (no blinding, no placebo possible). Up to now, no studies are available which have examined the reliability of the diagnostic tool “sting challenge test” [10]. On the other hand, unlike the situation during a field sting, sting challenge tests are highly artificial: contraindications for participation in a challenge test are observed, the patients are sober (no alcohol), and physical exertion is avoided. On the other hand, the patients know what to expect and are more or less nervous. 

In this study we aimed to investigate the prognostic value of a tolerated sting challenge with regard to future field stings. 

## Materials and methods 

This retrospective study was done in the AllergieZentrum of the Hospital of the University of Munich, Germany. The local institutional review board had no objection to an anonymous retrospective review of the notes of patients (Nr. 485-16). Inclusion criterion was a tolerated in-hospital sting by that insect species whose venom had originally been responsible for the reported anaphylaxis. Some of the patients who tolerated the sting challenge test had been included in a cohort that examined the effectiveness of VIT [3]. A prerequisite for performing a sting challenge test was that the patient had been on maintenance therapy with at least 100 µg of the relevant insect venom for at least 6 months. The maintenance therapy (Alutard, ALK-Abellό, Hørsholm, Denmark) needed to be tolerated without experiencing systemic allergic reactions. Typical local reactions to VIT or to the insect sting were considered as tolerance with respect to a systemic allergic reaction. 

Of the 598 patients who underwent sting challenge between 1999 and 2003, 585 (97.8%) had no systemic allergic reactions and were therefore eligible to participate in this study. If a patient had tolerated a sting challenge, they were recommended to undergo yearly follow-up examinations until treatment stop. The duration of VIT was typically 5 years, but it was extended on a case-by-case basis for patients with risk factors (e.g., mastocytosis) or when VIT stop would have negatively impacted their quality of life. The follow-up examination was conducted while the patients were still on VIT and concluded for all patients no later than 2012. During those subsequent out-patient visits, the medical history was routinely collected using a standardiz ed questionnaire asking for possible field stings and their secondary reactions. We also measured venom-specific IgE antibodies and performed skin prick tests. 

## Results 

We aimed to include 585 consecutive allergic patients who had 598 tolerated sting challenge tests (102 bee venom, 401 yellow jacket venom, 82 bee and yellow jacket venom). 56.2% were female, and 3.8% had mastocytosis (22/585). Patients with anaphylactic sting reactions of grade I – III according to Ring and Messmer [11] before VIT were included. 19/22 patients with mastocytosis reported a grade III anaphylactic sting reaction prior to VIT. 

A completed questionnaire was available from 439 patients (75% of the total cohort; n = 585). The data for the group that fulfilled the inclusion criteria are summarized in Table 1. Out of 439 patients, 24.6% (108) had at least had one field re-sting (71 one sting, 22 two stings, 11 three stings, and 4 > four stings). Patients (58 males, 50 females, mean age 52.2 ± 15.2 years) had been re-stung between 1 month and 7 years (median 2.5 years) after the tolerated sting provocation. Two sting incidents had to be excluded because the patients had been stung by an irrelevant insect. The majority of patients had been stung by a wasp (74) and 25 patients by a bee (this includes 5 patients who had had stings by both species). One patient had been stung by a bumble bee, and 17 stings (8 patients in addition stung by bee and/or wasp) were caused by not clearly identified insects. Based on the overall situation, these stings were classified as probably caused by the disease-relevant insect. 

104 of 108 patients (96.2%) tolerated the sting without any reaction. Four patients (3.8%) reported symptoms after the sting; 2 of them reported unspecific symptoms (lightheadedness) right after the sting, which spontaneously subsided. These symptoms were classified as only subjective psycho-vegetative reactions. Only 2 patients (1.9%), however, reported symptoms that were classified as mild systemic allergic reactions (clinical data are presented in Table 2). There were no signs of mastocytosis in either patient. Both were elderly women with pre-existing internal diseases who were being treated with several medications, including antihypertensive drugs. 

## Discussion 

Due to the small number of affected patients, it was not possible to identify specific risk factors for the occurrence of systemic allergic reactions despite a previously tolerated sting challenge. It is noticeable, however, that both patients who developed a systemic allergic reaction to an accidental re-sting were on various co-medications treating multiple medical diseases. There was no indication of mastocytosis. 

Besides the limitations described above, two additional factors may explain the “falsely negative results” of the sting challenge in those 2 patients: a) We could not perform an entomologic identification of the insects inducing the systemic reaction after the field stings. Therefore, we cannot exclude the possibility that the patients had been stung by another type of Vespinae, e.g. *Dolichovespula*. Allergy to *Dolichovespula* venom, however, cannot sufficiently be treated by standard venom extracts containing *Vespula* venoms. b) It is known that reactions to insect stings also depend on individual patient-related co-factors [3], which may vary over time and which may not have been comparable between the time of the sting challenge and that of the field sting. 

We conclude that tolerated in-hospital challenge tests have a high negative predictive value for the outcome of later field stings at least during ongoing VIT (98.2%). For most patients, this also means that it is not necessary to carry an emergency kit after a tolerated sting challenge. Besides this clinical aspect, a negative in-hospital challenge test result will also be psychologically helpful by significantly improving the patient`s quality of life [12]. Finally, a tolerated sting challenge during VIT is also relevant for the long term-prognosis when treatment has been discontinued [8]. 

Only very few patients who have tolerated a sting challenge will subsequently develop systemic reactions after a field sting even if they had tolerated a sting challenge with the culprit insect and if they are under continued VIT. Patients with risk for severe reactions should therefore carry an emergency kit with them even after a well-tolerated insect challenge test. 

## Ethical statement 

The retrospective study was approved by the Ethical committee of the Ludwig-Maximilian-University of Munich (Nr. 485-16). Written consent was not required according to the approval. 

## Authors’ contributions 

NW interpreted data, wrote manuscript, EO interpretetd data, enrolled patients, contributed to the manuscript, JG collected and evaluted data, contributed to the manuscript, AW, SG contributed to the manuscript, FR conceptualized and performed the study, collected and interpreted data, contributed to the manuscript. 

## Funding 

There was no funding. 

## Conflict of interest 

EO, JG, AW, SC report no conflict of interest. NW received research funding from and/or is or recently was a speaker/advisor for ALK-Abelló, LEO Pharma GmbH, Novartis Pharma GmbH, Takeda, Blueprint, CSL-Behring, Biocryst, Synlab, Nutricia unrelated to the submitted manuscript. FR reports honoraria and/or consulting fees from ALK-Abelló, Blueprint medicines, Boehringer Ingelheim, and Thermo Fisher Scientific unrelated to the submitted paper. 

**Table 1.** Patients with field stings during ongoing venom immunotherapy (n = 106) after tolerated sting challenge (n = 439).

	**Patients with tolerated sting provocation (n = 439)**	**Patients with field stings during ongoing VIT (n = 108)**
Therapy with BV	74 (16.8%)	18 (16.66%)
YJV	298 (67.88%)	67 (62.03%)
BV and YJV	67 (15.2%)	23 (21.29%)
Men	193 (43.96%)	58 (53.70%)
Women	246 (56.03%)	50 (46.29%)
Mean age of men (years ± SD)	46.2 (± 17.3)	48.2 (± 16,8)
Mean age of women (years ± SD)	51.6 (± 15.4)	56.9 (± 11.5)
Classification of severity of anaphylaxis (Ring and Messmer) [11]		
I	67 (15.2%)	12 (11.11%)
II	276 (62.9%)	60 (55.55%)
III	96 (21.9%)	36 (33.33%)
Mastocytosis confirmed	22 (5.01%)	9 (8.33%)
No indication of mastocytosis	317 (72.20%)	99 (91.66%)

**Table 2.** Characteristics of patients with allergic immediate-type symptoms despite tolerated in-hospital sting challenge.

	**#1 (female)**	**#2 (female)**
Age at the time of the field sting	70	64
Sting symptoms prior to VIT, severity score according [11]	III	II
Venom	*Vespula*	*Vespula*
VIT dose	100 μg	200 μg
Interval of maintenance injections	Every 6 weeks	every 4 weeks
Duration of VIT until field sting	68 months	34 months
Interval between tolerated sting challenge and field sting	3 years	1 year
Symptoms at field sting (severity score according [11])	Dizz, W, E, AE (II)	D, E, P (II)
Treatment of symptoms	Application of emergency kit	Medical treatment not known
Medication at the time of the field sting	Verapamil, allopurinol, triamterene, HCT, ASA, atorvastatin, risedronic acid	Simvastatin, levodopa, benserazide, candesartan, HCT, clopidogrel, metformin, budenoside/formoterol
Special features of history, findings	BTC: 5.6 µg/L	BTC: 3.29 µg/L SAR at first sting challenge following VIT with 100 µg; SAR at the subsequent dose increase (up to 200 µg); 2^nd^ sting challenge following VIT with 200 µg had been tolerated

VIT = venom immunotherapy; U = urticaria; P = pruritus; E = erythema; AE = angioedema; D = dyspnea; W = weakness; dizz = dizziness; ASA = acetylsalicylic acid; HCT = hydrochlorothiazide; BTC = baseline tryptase concentration; SAR = systemic allergic reaction
